# Standing Still at Full Speed: Sports in an Overheated World

**DOI:** 10.3389/fspor.2021.678987

**Published:** 2021-05-14

**Authors:** Thomas Hylland Eriksen

**Affiliations:** Department of Social Anthropology, University of Oslo, Oslo, Norway

**Keywords:** competition, skiing, sustainability, evolution, treadmill

## Abstract

In evolutionary biology, the “Red Queen Effect” refers to a form of inter- or intra-species competition where continuous improvement is necessary in order to survive and thrive, since the other species/individuals evolve. In sport, the same mechanism can be easily observed, and this article explores its implications. It discusses improved training regimes, scientific diets, innovative techniques enhancing performance, and technological improvements such as fibreglass skis. It argues that the upward spiral of improved achievement can be seen as an effect of the global market, or of the modern values of development and growth which are celebrated in modern sports. The world of competitive sports is not just an integral part of global capitalism, but it also mirrors and mimes its internal logic. The kinship between sport and war is obvious, and many sports grew out of military training. But since much of the world has been spared the horrors of war for generations, in the very same period that capitalism has become ever more hegemonic and globalised, sports in the 21st century have come to resemble market competition more than bloody events on the battlefield. Not least for this reason, the treadmill paradox, or Red Queen effect, easily discernable in market economies as a driver for change, whether progressive or destructive or both, can fruitfully be applied as an analytical lens through which to view sport. The question nevertheless remains to be answered as to whether the improved achievements of athletes lead to an improved spectator experience or the opposite. In this question lies an inherent paradox of contemporary world civilisation, with a literal as well as a metaphorical bearing on the critique of the unsustainable growth economy.

*“Well, in OUR country,” said Alice, still panting a little, “you'd generally get to somewhere else—if you ran very fast for a long time, as we've been doing.”*

*“A slow sort of country!” said the Queen. “Now, HERE, you see, it takes all the running YOU can do, to keep in the same place. If you want to get somewhere else, you must run at least twice as fast as that!”*

*“I'd rather not try, please!” said Alice*.

—Lewis Carroll: *Through The Looking-Glass*

## Introduction: The Treadmill Conundrum

In evolutionary biology, the Red Queen Effect refers to a particular aspect of competition (Van Valen, 1973) or a long-term result of sexual selection. As in the situation involving Alice and the Red Queen, an organism, or species, is forced to evolve continuously merely to survive, since its competitors, prey or predators evolve. As rabbits become faster, foxes have to follow suit in the *longue durée* of evolution. An organism has to evolve merely to retain its niche, simply because its prey, predators or competitors evolve. In Ridley's (1993) account, the emphasis is on intraspecies competition. If you are a young spruce in a dense forest, you will have to grow to a height of 20 or even 30 m in order to absorb enough sunlight to reproduce, since you are surrounded by trees of roughly the same height as yourself. There is no immediate evolutionary or individual advantage in this competitive race, which is why I propose to call it a treadmill syndrome. The trees, one might say, would have been just as healthy and happy if they had settled for a height of 5 or 6 m; what forces them to grow taller is the height of their neighbours, and the taller tree produces the most acorns, thereby spreading DNA programming its offspring to grow tall as well.

Although it may look like an accelerated standstill, treadmill competition drives evolution, forcing species and individuals to improve their achievements relative to others over the generations. The “competitive edge” often invoked in technology and business refers to a quality enabling a company, product or activity to “edge” ahead of the others, who will in turn have to follow suit. There is a resemblance, in other words, between the cheetah evolving greater speed to catch gazelles who are nimbler and faster than their ancestors, and mobile phone developers looking to eclipse their competitors with a sleeker design, better camera or larger screen.

One may ask why this model from evolutionary biology should be applied to processes taking place in society. There is widespread wariness toward applying evolutionary models to the social sciences, often with good reason. Yet, treadmill phenomena are so easily identifiable in social life—in sport, fashion, technology, academic publishing, just to mention a few examples—that the pattern resemblance with events on the savannah, or in the forest, is nothing less than striking (Hessen and Eriksen, 2012 is a book-length exploration, in Norwegian, by an anthropologist and a biologist, of this phenomenon). A main difference between cultural and evolutionary treadmills is the fact that humans can decide to do things differently if the spirals of the treadmill threaten to become destructive. One characteristic of the globalised resource economy is nevertheless the lack of an actor, a governor or thermostat (Eriksen, 2016), which would have been capable of regulating growth and slowing change down when necessary. Like industrialists or investors, the decision-makers in the world of competitive sport have no choice but to play according to the rules, trying to gain those extra inches enabling them to catch more sunlight, as it were, than their close neighbours. While this form of competition is integral to capitalist growth and what is known as progress, it is also, as a guiding principle for life and economic activity, a recipe for ecological disaster. It encapsulates, in a nutshell, the double bind of contemporary industrial capitalism, suspended in mid-air between growth imperatives and a desire for sustainability.

The world of competitive sports is not just an integral part of global capitalism, but it also mirrors and mimes its internal logic. The kinship between sport and war is obvious, and many sports grew out of military training. But since much of the world has been spared the horrors of war for generations, in the very same period that capitalism has become ever more hegemonic and globalised, sports in the 21st century have come to resemble market competition more than bloody events on the battlefield. Not least for this reason, the treadmill paradox, or Red Queen phenomenon, easily discernable in market economies as a driver for change, whether progressive or destructive or both, can fruitfully be applied as an analytical lens through which to view sport.

Indeed, it can be said that the treadmill principle is scarcely more applicable to any other phenomenon than competitive sports, and it is pithily, if inadvertently, epitomised in the Olympic motto *Citius, Altius, Fortius*—Faster, Higher, Stronger. A group of anthropologists who studied a major sport event as a ritual (Klausen, 1999), were faced with a conundrum. Doing an in-depth study of the Winter Olympic Games at Lillehammer in 1994, they drew on earlier anthropological research on rituals in non-state societies for inspiration. However, these rituals typically were devoted to transitions between life stages (Turner, 1969; Bloch, 1986), religious transcendence (Bell, 1997), the glory of the nation (Kapferer, 1988; Connerton, 1989) or competitive masculinity (Geertz, 1973). The event under scrutiny certainly had elements of several of these, especially nationalism and masculinity, but it did not quite seem to fit. In the end, the research group agreed that this particular ritual was a celebration of modernity—speed, efficiency, growth for growth's sake without an ulterior aim. It went without saying that faster was better.

The similarity with Aldous Huxley's *Brave New World* is striking. In his famous 1932 novel, he depicted a consumerist, hedonistic society which had forgotten the art of storytelling, where tragedy had been erased from the cultural repertoire, and where the only acts of worship were the regular songs of praise to Ford (a cheap, but irresistible pun on “Lord”).

The phenomenon itself is well-known, but not sufficiently theorised: It doesn't help that your team improves if the other teams improve more. Your achievement is relative to that of others. None of the finalists in the 100 m dash at the Seoul Olympics in 1988 would have qualified 20 years later. If the competitors acquire swimsuits which reduce friction (or lighter and stronger hockey sticks, or football boots which fit like a second skin), you have to emulate, if possible with a slightly superior solution.

Treadmill competition is tacitly known and acknowledged across the world of competitive sports. As some of my examples will show, the spiral of improvement through competition is often inherently environmentally destructive. A more difficult question, which I will also raise, concerns whether the spectator experience has improved. Is speed skating more fun to watch now than it was in the 1960s? It certainly was more popular, in countries like the Netherlands, Sweden and Norway, at that time. Has football (soccer) become more entertaining to watch now that the speed, precision, fitness and technique of the players is immeasurably superior to that of the previous century? The question cannot be answered unequivocally, but it deserves to be asked.

## Treadmills in Nordic Skiing and Beyond

Winter sports, unfamiliar in most of the world, have a substantial and commercially important niche in the affluent, cold countries, although the popularity of the different sports vary. Curling is huge in Canada, but marginal in Finland. Alpine skiing is popular in the Alps, less so in Sweden, where ice hockey is a national sport. Cross-country skiing, although practised across the cold regions of the planet, is nowhere else nearly as popular as in Norway, and unsurprisingly, Norwegians tend to win most of the medals in international competitions. Nordic skiing is also an important element in Norwegian national identity, and is practised recreationally by a large proportion of the population.

Teams of specialists, funded by the Norwegian state, ensure optimal conditions for the elite participants. A busload of ski wax, machinery for preparation and fine-tuning and about a dozen dedicated waxers accompany the national team wherever it goes, and the science of waxing has developed rapidly. In the 1960s, a photo was taken of the elite skier Harald Grønningen ahead of the 50 km race at Holmenkollen in the forest above Oslo, applying wax to his pair of wooden skis. Soon after, waxing was done not by the skiers themselves but by specialists, and in the 1970s, wooden skis were replaced by fibreglass skis, amidst controversy as purists continued to vouch for wood until they saw no option other than converting to the superior material. As to waxing, chemistry professors have been involved as consultants, analysing the relationship between the quality and temperature of the snow and the wax, or sticky wax, or powder, or glider, to be applied to the skis. This research, aimed to improve the performance of the national team, has an air of secrecy comparable to that surrounding lab tests of coronavirus vaccines or nuclear experiments. However, the competition has no choice but follow suit, lest they are left behind. As the Red Queen says to Alice, you have to run as fast as you can to stay in the same place. Today, even junior skiers at the elite level need to have several pairs of skis and boots (for classic and skating races), a cupboard full of wax and related products, an iron and a tuning kit, plus brushes, cork and a few more implements. When I was a boy half a century ago, the typical child had one pair of skis, and their family would have at their disposal four tubs of wax for different temperatures and a square cork for applying the wax, doubling as a scraper to remove old wax. While this modest adaptation remains widespread, the proliferation of specialised gear is perceptible to anyone who enters a contemporary sports shop: It is sufficiently large to require a map for orientation, and the amount of skiing equipment for sale is extraordinary in its quantity and diversity.

New methods for training are continuously developed along with aerodynamic, functional clothing, optimal diets and scientifically based tactics, comparable to that of the battlefield. Records are continuously broken, yet there can only be one winner and a total of three medallists. For every winner, there are many losers, and the relationship of winners to losers is constant regardless of everybody's improved performance. Since competition is relational, the performance of the others is crucial, and their slump is your good luck. This, too, is constant. Within the group (which could be a team of individual athletes), there may be team spirit, solidarity and mutual support (even if sometimes ambiguous); between groups competing for the same scarce resource, there is by default competition. As the evolutionary biologist David Sloan Wilson (2015: 71) puts it: “Selfishness beats altruism within groups. Altruistic groups beat selfish groups. Everything else is commentary.”

Yet, in accordance with the growth imperative of capitalism, the general tendency is that of improvement, which is easy to measure in the stopwatch-based sports, more difficult in team sports. Yet, it is exceedingly likely that the 2020–21 version of Manchester United, more than decent but trophyless, would have beaten the 1998–99 team, which won the triple of the Premier League, the FA Cup and the Champions' League in an unprecedented demonstration of power. Similarly, every participant in the 2021 speed skating World Championship would have won the 1965 edition of the same event.

Treadmill competition drives technology, training regimes, equipment, and selection pressures forward. Once upon a time, an exhilarated journalist exclaimed that 15.46.6 would be an unbeatable world record. That was the speed skater Knut Johannesen's finishing time at the 10,000 m race at the Winter Olympics in Squaw Valley back in 1960. He crushed the previous record with almost half a minute. 50 years later, Lee Seung-Hoon won the same distance with a time of 12.58.55. The best Norwegian, Håvard Bøkko, ended in fifth place with a time that would have beaten Johannesen with four or five rounds out of a total of 25. The current (2021) world record for the 10,000 m distance is 12.32.95 (held by the Swede Nils van der Poel). However, speed skating, a famous and important sport in a handful of countries a couple of generations ago, has disappeared nearly completely from the public eye. The niche has shrunk.

Competition becomes uninteresting for everyone if the gap between the best and the rest is too wide. In Norwegian football, this was the situation for years, owing to the fact that Rosenborg was far better than the competition. With a couple of exceptions, they had won the domestic league every year between 1992 and 2010. In a competitive setting like this, the superior team is likely to stagnate, while those immediately below improve through competing for second place, recalling Hegel's master–slave (actually lordship–bondage—*Herrschaft und Knechtschaft*) dialectic: The master has no incentive to improve, whereas the slave aims to improve in a bid to emulate the master. The best team is exposed to few reality checks revealing its incipient deterioration. And indeed, in the last decade, Rosenborg has struggled, whereas others have won the domestic league and enjoyed moderate success in Europe.

A comparable situation arose just as I completed the previous paragraph. Since cross-country skiing has been dominated by Norwegians for a number of years, competition is dwindling, and recruitment to the sport in other countries suffers. When, on 6 March 2021, a Norwegian woman once again won a race in the World Championship in Nordic skiing, the German coach remarked that the predictability of results took the fun out of the competition, going on to intimate that there was something unnatural about the extreme achievements of this particular Norwegian athlete. He added that there ought to be a BMI limit for performers, noting that some of the Norwegians were uncannily skinny. Predictably, the Norwegians reacted with disbelief and outrage, but it is not irrelevant that the athlete in question (Therese Johaug) lost two seasons owing to a controversial, and contested, positive test for illegal substances. In international competitions, nationalism adds considerable fuel to the treadmill since athletes are depicted, by domestic media, as somehow representing the collective will of the entire nation.

## Technological Innovations

As noted, technical innovations change the conditions for competition. The introduction of spikes made an enormous difference for track runners after their introduction by English athletes in the 1920s, the author and inveterate runner Coles (2016) detailing not only the invention of spikes, but also other shoe storeys with important consequences for sport, not least delving into the competitive relationship between the Dassler brothers Adolf (Adi) and Rudolf (Rudi), who would found Adidas and Puma, respectively. In cross-country skiing, the last major performer using wooden skis was Magne Myrmo, who won the 15 km race at the World Championship in Falun in 1974, narrowly beating Gerhard Grimmer, who was on fibreglass skis. Since then, nobody has won anything on wooden skis.

A decade later, the American Bill Koch initiated a new skiing revolution by introducing the skating technique (also known as freestyle). Seen in a broadly evolutionary, or perhaps capitalist, perspective, this innovation represented an improvement, which retrospectively appears almost as inevitable, since the tracks were now sufficiently broad and well-prepared for a new niche to be filled: Using the skating technique enabled faster racing than the classic style, since the tracks were by now not surrounded by loose snow. Although it was not banned, the technique was controversial. Similar to the situation when fibreglass skis appeared, there was lively discussion as to how to deal with this new technique. However, although puritans regarded the skating technique as ugly and inelegant, it was difficult to turn the clock back. Skating went through its own accelerated microevolution and was perfected, and in terms of speed, it was sufficiently superior for “classic” and “skating” to be separated in competitions. Naturally, from now on, skiers needed twice as many pairs of skis, poles and boots. Plus equipment for preparing the new skis.

Skating was contested, but a more toxic scandal erupted when the Swedish ski jumper Johan Boklöv introduced a new style, known as the “V style” or simply the “Boklöv style,” of jumping. Instead of keeping the skis parallel during the gliding journey from ramp to bottom of slope, hitherto seen as indication of elegace, he held the skis diagonally in a 45 degree angle, in an aerodynamically superior shape that enabled longer jumps. Ski jumping is unusual in that it, like figure skating, involves style scoring, and in the beginning, Boklöv and his supporters may have lost a few style points, but they were compensated by superior length. This is no longer an issue, as the V style eventually became the norm.

With ski jumping, another form of treadmill competition is also at work, concerning the height of the ramps. The first ski jumping competition at Holmenkollen was held in 1892. Arne Ustvedt won the prize with a 21.5 m long jump. Since then, the hill has been rebuilt and expanded many times, and the record has been improved on more than 70 times, most recently by Robert Johansson, whose 2019 jump measured 144.5 m.

Ski flying is a sub-branch of ski jumping, where length is everything. There are just five ski flying venues, all in Europe. At the time of this writing, the world record is held by Stefan Kraft with 253.5 m. However, the hills are being upgraded, and when Vikersund (Norway) expands, that is a strong incentive for Planica (Slovenia) to do the same, even if it means that the Slovene state may have to relinquish some services for their elderly or schoolchildren, and even if the ultimate benefit is debatable. On the other hand, as sport executives and governments (their primary funders when it comes to infrastructure) are aware, unless ski jumpers can show progress, they are bound to lose attention and sponsor funding. In this particular case, there nevertheless seems to be a thermostat, or governor, limiting the otherwise perpetual growth. Above a certain limit, ski flying would become too dangerous. Wind is already an issue in normal ski jumping, leading competitions to be cancelled if the risk is deemed to high; with ski flying, even a mild breeze could be sufficiently hazardous to result in serious damage on a bad day.

It may be tempting to look at treadmill competition—standing still at an increasing speed—as a simple zero-sum game where the defining relationship remains constant because all the components of the system change roughly at the same time and speed. However, there is also a material side to treadmill competition. The upgrading of the Holmenkollen and Vikersund hills in the early 2010s was very expensive (in the region of €180 million and €10 million, respectively), and from an environmental point of view, none of this can be said to be healthy. Similar examples are easy to find. The now infamous football stadiums being built in Qatar, about which the tired word “spectacular” has probably been used millions of times, are meant to outshine stadiums in other countries in terms of functionality and aesthetic qualities. Stadiums have been upgraded, refurbished and even demolished elsewhere as well. As a child, I was taken to Highbury to watch Arsenal with my father and older brother. I still remember the faint smell of urine, the cigarette smoke, the having to stretch your neck to see any of the action since everybody was standing, and not least the reddish faces next to us screaming for penalties with such an intensity that we had to pull out our hankies to wipe off the moisture. It was a powerful and memorable experience, still lingering in my olfactory memory. A generation later, I took my son to Emirates to watch a very different (doubtless much better) Arsenal team; all surfaces were slick and clean, and as we slumped down in our comfortable seats, we noticed that the people next to us were sober and well-behaved. The event as I remember it was a tepid and boring experience (and that was not just because the Gunners failed to beat Chelsea).

## Ambivalence to Technological Innovation

In the world of cross-country skiing, the *Fédération Internationale de Ski* (FIS) took a somewhat anxious and ambivalent attitude to the innovations which emerged at the exact same time that neoliberalism became the dominant global ideology, that is the 1980s. Eventually, they bowed to the market of sponsorship, lucrative television rights and public interest, not only accepting the Boklöv style and skating, but embracing every innovation that could raise greater public interest through more intensive, compressed events, shorter distances, simultaneous starting (in the past, there would be 30 s between each performer) and a more commercial feel in order to keep the place of the sport as such on the treadmill and not succumb to the same sad fate as the 10,000 m ice skating distance, which virtually disappeared as the acceleration of multi-channel entertainment began to take off (see Eriksen, 2001, 2016 about acceleration in general).

Treadmill competition leads not just to acceleration within each sport, but acceleration of sport as such. The archetypal cross-country skier a generation or two ago was a quiet man who had little to say when interviewed, and during speed skating championships, spectators had to suffer through a few uninteresting pairs (they race in twos), colloquially known as *buljongpar* (“broth pairs,” meaning that you could in good conscience go and get a hot savoury drink when they ran), during the final and often decisive 10,000 m distance. These forms of slowness have been removed for the sake of speed, efficiency and progress, and owing to intensified competition for audience attention. There was nothing inherently superior about wooden skis with just one layer of purple wax and taciturn fellows who disappeared into the forest for an hour or two before emerging on the stadium, as opposed to the slick, efficient, finely tuned performers who now race in the continuous floodlight of cameras and electronic timing systems, but it may have been just as good. It may well be the case that audiences are no less enthusiastic today—regardless of which sport—but possibly not more either. Speed, dramatic highlights and entertainment value are forced to grow in an ecology of entertainment where the attention of the audience has become the scarcest resource. The net result is that both the athletes themselves, the support system engulfing them, the sponsors who own the athletes and the institutions framing the sports, have to run like hell just to keep their place.

I have spoken of cross-country skating, the V-style and fibreglass skis. Perhaps it speaks of the conservatism or relative insularity of Norwegian winter sports that Norwegians have never proposed any of these innovations, but indeed resisted them. This happened—for a fourth example—when the clap skate was introduced by Dutch ice skaters, also in the 1980s. Unlike traditional skates, the steel of the clap skate is attached to the boot through a hinge at the front, enabling more flexible and dynamic foot movement and greater overall speed. The Norwegians immediately wished to ban the clap skates because they made for unequal competition. And yet: After a few years, the Norwegian Ådne Søndrål won a world record on clap skates, and since then, nobody has looked back.

Several of my examples have shown how technological innovations change the conditions for competition, and when someone innovates, others are forced to follow. A typical argument against divestment from fossil fuels is that a single country cannot make a difference, and as long as others use and produce fossil fuels, it would be foolish—a self-imposed handicap—not to do it oneself. The typical argument in favour of frequent flying is that the plane takes off anyway, so my input makes little difference. This kind of argument shows how neoliberal thinking has become second nature: According to this world-view, societies do not exist, and changes can only be made to happen through consumer choices. There seems to be no instance at a higher scale which can regulate, slow down and scale down the subsystems of globalisation in order to make them more sustainable. In the world of competitive elite sport, ecological sustainability is barely on the agenda, and technical innovations, specialisation and intensified competition, as witnessed in ever more frequent international tournaments, is consistent with the treadmill principle, where the spiralling growth assumed to lead to progress (but which may *de facto* lead to everybody standing still at full speed) requires increased consumption, infrastructural developments, incessant travel (with a growing support system in tow) and sometimes direct environmental destruction, as when those 30 m tall spruce trees in the hills around Oslo are unceremoniously removed to make space for wide ski tracks enabling easy monitoring and not least filming for the benefit of TV viewers, whose habits have changed, in the space of just a few years of intense competition for their attention, such that live sport events, including war reporting from the frontline, are about the only forms of programming that make sense as linear television.

In some sports, technology is less important than technique. In football, the by far most popular sport in the world, clothing is of little significance (shorts lengths have followed fashion, not functionality; historically, the smallest shorts were those worn by the Argentine national team at the 1978 World Cup, and in the following years, the hem began to move down the thighs again, eventually reaching the 1970 length); football boots are admittedly of some significance, but the most important changes have concerned technique and strategy. When Pelé, in the 1950s, showed the bicycle kick to the rest of the world (in Norwegian it is till this day known as *brassespark*, “Brazilian kick”), stiffer players from the Protestant North began to practise shots on goal which required them to throw their body backward while kicking the ball across it and, with luck, into the GOOOOOL. Similarly, Johan Cruyff's success with “total football” at Ajax and later with Barcelona led other football coaches to experiment with a team structure where any outfield player could in principle swap positions with any other player.

## Niche Production

Allow me to return to winter sport. The by far best speed skater in the late 1970s was the American Eric Heiden. In 1980 he won all five gold medals at the Winter Olympics, as the first and last skater to do so. Aware that there remained no challenges for him in this—globally speaking—tiny sport, he quit ice skating and took on bicycle racing. This is a far larger sport where competition is tougher, and he never won anything more significant than an American championship. Yet it seems as if Heiden preferred to be a small fish in a large pond. Rosenborg and Heiden have something in common, but the former cannot switch to a larger and better league in the hope of improving (In the more porously bounded British nations, suggestions have been made to the effect that Celtic and Rangers join the English Football League).

Others move in the opposite direction, exploiting niches where competition is less fierce. In some of the highly competitive, quantitative, individual sports, margins are tiny. Take swimming, where there are rules regulating the nature of the swimsuit, but when, in the 1980s, some swimmers began to remove all body hair, others followed, even if the actual gain could be just a few hundredth of a second. As with the 100 m dash, the significance of a few centiseconds indicates a crowded field hard to squeeze into. Some might be well-advised to abandon swimming for orienteering or decathlon.

Not all ideas have yet been thought, and not all tunes have been performed. There are still niches to be filled, or perhaps more accurately to be carved out and created. In sport, new niches and their accompanying treadmills are continuously being introduced, leading not least to the increasing visibility of women in sports traditionally regarded as inherently masculine, such as boxing, football and ski jumping.

Sport differs from other competitive arenas. It is said that “all's fair in war and love,” but in sport, this is not the case (There are rules regulating war too; poison gas is not tolerated, while massacres of civilians are). In the competitive arena of romantic love, on the other hand, anything goes; doping is fine, whether it appears in the shape of pills, whisky, botox, silicone, lovely clothes, dyed hair or makeup, or if declarations of love are conjuring tricks with the ultimate, but hidden aim of getting laid. In sport, there is a relatively well-defined boundary which forbids the use of performance-enhancing stimuli—but on the other hand, improvements within the realm of diet, gear and support systems, technique and technology are often permitted. There are rules limiting the freedom of technological developments, regulating anything from the fabric and fit of skijumpers suits to the studs on football boots. Sometimes, rules can be circumvented and sometimes they may be stretched, but there are real sanctions applied in the case of blatant violation. On the other hand, competition is often skewed in sports that rely on extensive gear, but this is difficult to change. Had it been easy, the powers that be would long ago have decided that all entrants in cross-country skiing competitions should have identical skis waxed in identical ways.

The next question is not whether treadmill competition is bad for the environment (which it is), but what it tells us about current and hegemonic views of progress. It is as if modernity has shifted into a higher gear (Eriksen, 2016) since the onset of global neoliberalism, prefigured in the historical persons of Margaret Thatcher and Ronald Reagan, and flourishing after the end of the Cold War around 1990. Ours is a world of high-speed modernity where the fact that things change no longer needs to be explained by social scientists; what comes across as extraordinary or puzzling are instead the patches of continuity we occasionally discover. Modernity in itself entails change, but for decades change was synonymous with progress, and the standard narrative about the recent past was one of improvement and development. Things seemed to be getting better, and history had a direction.

In the last few decades, the confidence of the believers in unilinear progress has been dampened. Modernity and enlightenment did not eradicate atavistic ideologies, sectarian violence and fanaticism. Wars continued to break out. Inequality and poverty did not go away. Recurrent crises with global repercussions forced economists to concede, reluctantly, at least when caught with their pants down (e.g., during the 2007–08 financial crisis), that theirs was not a precise science after all. Although many countries were democratic in name, a growing number of people felt that highly consequential changes were taking place in their lives and immediate surroundings without their having been consulted beforehand. And, most significantly, the forces of progress turned out to be a double-edged sword. What had been our salvation for 200 years, namely inexpensive and accessible energy, was about to become our damnation through environmental destruction and climate change.

This is the broader horizon on which we must see the treadmill syndromes and paradoxes of competitive sports. *Citius, altius, fortius* was a forward-looking and promising ideal at a time when there were <2 billion of us and the world was open, in the decades preceding the First World War, to utopian projects, be they liberal, socialist or anarchist. The first modern Olympic Games, in Athens, took place in 1896, when the total world population stood at 1.9 billion. Ours is a different world, where the free flow of the nearly empty highway has been replaced by a busy road on the verge of a traffic jam, where the shadows of environmental destruction, inequality and climate change dampen enthusiasm and indeed the belief in progress as such.

Let us, true to character (since the pandemic has taught us to live locally, even if we may still occasionally think globally), return to the nation-building Norwegian pastime and competitive sport of cross-country skiing. Today, people may chuckle at old images from ski races from around 1915, when strapping young fellows, none of them professional athletes, but holding day jobs, often as lumberjacks or farmhands, vanished into the forest on badly prepared tracks, in knickerbockers and woollens, on well-worn wooden skis. On the way, they would stop by a soup station and nod congenially to the supporters cheering them on along the track, before reapparing, leaden after 50 km of hard skiing, at the stadium where they would be rewarded by the sight of an ecstatic crowd. A century later, leading athletes would race through the same forest, but this time on a 10 m broad highway, professionally prepared with the aid of dedicated machinery including chainsaws, every gliding movement recorded by a camera, typically completing the race in a little over 2 h.

The anthropologist Odd Are Berkaak has characterised the ethos of competitive sport as “larger than large” (Berkaak, 1999). Not only the extreme physical achievements of the athletes themselves are worshipped, but also the growth ideology of accelerated and accelerating modernity. Nobody would care to watch a cyclist or runner who is slower than last year's performer. A reduction in speed and efficiency would be deemed unacceptable. Recall that some of the major technological innovations in winter sports were introduced precisely at the time when neoliberalism was on its way to global hegemony, and were consolidated in the upbeat years following the end of the Cold War. It may well be asked whether this ethos will continue unscathed in the coming years, as the unsustainability of the growth economy has become widely known and acknowledged, and where degrowth economics (Hickel and Kallis, 2020) is becoming a force to be reckoned with. In sports, the physical limitations of humans may be a ceiling comparable to the carrying capacity of the planet. Perhaps genetic engineering will solve the problem, but it is worth pondering that the 100 m world records have been held by Usain Bolt since 2009 and by Florence Griffith-Joyner since 1988. Bob Beamon's sensational long jump in 1968 (8.90 m) remains, more than half a century later, the second-longest jump ever recorded. A ceiling may be approaching. It would have been the source of some relief if actors in the field of resource exploitation and economic planning saw theirs.

## The Evolving Mediascape

So far, I have concentrated on treadmill competition in the realms of technology and technique, strategy and infrastructure. However, the mediascape is also changing along related lines. Since commercial appeal is essential for the viability of any competitive sport, the ability to attract large audiences is subject to ever intensifying competition in a densely populated media world where channels and platforms proliferate. Even in the conservative world of cricket, where test matches typically last for 4 or 5 days, compressed one-day test matches have tentatively been attempted. Commentators, whether live at the venue or through a broadcasting channel, compete for attention by using dramatic effects. An early instance of this development was the legendary (in Norway) journalist Bjørge Lillelien, whose radio reporting from a 1981 football match where Norway beat England has entered the annals of national sport history. A sober and descriptive reporting style was now gradually giving way to high-pitched voices and ecstatic exclamations, and immediately following the final whistle, Lillelien extemporised a rallying cry invoking Lord Nelson, Anthony Eden and Winston Churchill, among others, switching briefly to English with the comment “Maggie Thatcher, can you hear me?” toward the end.

Both language and style have evolved toward hyperbole and overheating since then, the most striking example perhaps being that of Spanish-language football commentators when they cry “GOOOOOL!.” The law of diminishing returns nevertheless kicks in as a result of this kind of escalating, frantic search for attention. The word “incredible” (or *increible, incroyable* or *utrolig*) no longer means incredible, but rather or quite (Usage also changes in other contexts related to sport. Nobody wants to be a jogger any more; they are runners, often possessing gear far beyond that which is necessary to stay reasonably fit).

The proliferation of platforms and channels mentioned above is an important causal factor in these changes. The parallel with the intensified treadmill competition operating both within and between sports is evident. For capitalism to thrive, it has to grow, either by conquering new markets or by intensifying consumption in existing ones. In the case of spectator sport broadcast in the media, both strategies are visible. If we look at football (soccer), the world's most popular sport measured both by player numbers and spectators, the availability of satellite television has led to its expansion into new markets. As a result, several African football leagues struggle to fill their stadiums now, since many fans prefer to stay at home and watch their favourite European teams instead, often with more than a sprinkling of African players. Regarding the competition between sports, football has led to a significant reduction in diversity (with the USA as an exception, see Foer, 2006), as smaller sports have successively lost audiences and performers in the last decades (Eriksen, 2007), mirroring the increasingly dominant market position of Amazon in books and other consumer goods and, referring back to an earlier period of free-market capitalism, confirming Marx's analysis of monopoly capitalism in the Victorian age.

In the evolution of ecosystems, species about to be outcompeted by stronger adversaries may develop specialised niches—they may become scavengers, diurnal hunters, thornbush feeders etc. As noted, this mechanism also applies to sport, where national or regional sports such as hurling (Ireland), Aussie Rules Football (Australia), and Nordic skiing (Scandinavia) remain able to defend their turf. Smaller-scale regional sports may on the whole be more ecologically sustainable than the global ones, since they are less economically important, entail less travelling, more sensible payments and a less expansive infrastructure. This is nevertheless not necessarily the case, as the example of Nordic skiing indicates. The magic of football lies in part in its democratic appeal and cheap entrance ticket. There are global megastars in this game who grew up kicking a rubber ball barefeet in back alleys.

## What of Sustainability?

I have showed that there are several forms of treadmill competition taking place in sport, some of them overlapping and mutually reinforcing, such as market economics and nationalism. Leaving recreational sports aside, which have their own treadmills (and other aspects as well, such as improved health), we have considered technological advances, the mediatised competition for attention, the scientific fine-tuning of athlete bodies, competition between sports and within sports for glory, other people's attention and money, infrastructure and tactics. What they all share is an implicit commitment to the principles of the capitalist economic system, notably enhanced efficiency, growth and progress. I have suggested that the entertainment value on the part of the spectators may be constant (although such qualia cannot be measured), yet as Alice learned from the Red Queen, you have to run as fast as you can to stay in the same place. Change is the only constant; change is necessary to preserve continuity. Only three medals are awarded in individual sport, and only one performer or team wins the first prize.

Can escalating treadmill competition in sport be compatible with ecological sustainability? The question resembles the issue of green growth, or “decoupling” growth from environmental destruction.

The answer is that it depends on a number of factors (I would say that, now wouldn't I?). Some forms of treadmill competition have doubtless led to genuine progress and better lives. Think of the evolution of ordinary things, for example, such as the fork, the watch or the paper clip (Petroski, 1992). It is unlikely that they would have found their present, functional form through state planning. Other forms of treadmill competition are ecologically destructive, increase social inequality and have few net positive effects, such as the mammoth infrastructural project of building extraordinary football stadiums in the hot desert country of Qatar. Yet others are physically debilitating to individual competitors, and this is the case with various sports, whether they may lead to brain damage, anorexia or chronic pain. Athletes may, in a different argument, be likened to Roman gladiators, sacrificed by the mediatised, jaded and often nationalist public sphere, not to the gods or the Emperor, but to the spirit of modernity, losing their youth to ultimately futile and unhealthy exertion.

The simplest treadmill category is that in which two or several actors compete to take the lead relative to the others, typically when competing for a specific resource or niche, and where the costs and benefits generally are relevant just for those directly involved. Competition for a common, but renewable resource may fit here, and certain aspects of competition within and between sports fall into this category; audiences are renewable resources. However, in most cases, a third party will be affected, positively or negatively: The upgrading of football stadiums in the UK from the 1980s onwards reduced hooliganism, but tickets became too expensive for workers on modest salaries. The scramble for minerals, including hydrocarbons, has led to a phenomenal economic growth rate worldwide since the 1980s, at the expense of undermining the conditions for the civilisation created on the back of hydrocarbons. Besides, an initially positive outcome of a competitive race, such as cheaper commodities and services, may eventually lead to a reduction in diversity (standardisation, eonomies of scale) and of quality. An effect of cheap food is intensified exploitation of food producers, increased use of antibiotics, pesticides and chemical substances enabling the food to keep longer. A similar argument is often made with regards to garments. Treadmill competition, while increasing production and consumption, can thus turn into a race to the bottom.

What about sport? Many of the features of contemporary elite sport can easily be pinned down as accessories of global neoliberalism: the mediatised globalisation of events, leading to a growing gap between performers and audiences; the economic power wielded by the major actors; the infrastructural changes facilitating efficiency and maximal commercialisation; the technological and scientific races for superiority; and last, but not least, the commitment to growth and incremental improvement year by year. On the other hand, sports have always been competitive, and the young men (usually) who win prizes have always been celebrated as unique individuals, from Melanesian mock-fights to mediaeval jousting, from the Olympic games in ancient Greece to the fencing duel. Competitiveness is a product of evolution, as is its complementary quality, cooperation.

It would thus be a waste of time to attempt to create a socialist man once again, a human who has somehow evolved beyond competitive urges, vanity and one-upmanship. In addition, there is a demonstrably social element in spectatorship, as sociologists of sport have documented comprehensively (e.g., Dyck, 2000; Giulianotti, 2015). It could be said that football fandom has all the positive qualities of Fascism and few of the negative ones. As a true fan at the stadium, you worship the superhumans on the pitch and lose yourself as an individual in a mass of likeminded spirits waving scarves, chanting, cheering and booing. Yet after the match you feel no urge to go out and kill Jews or set fire to the houses where the fans of the opposing team live. With international competitions, the situation is more complicated, since the nationalism fanned via the medium of athletes wearing the national colours has severe negative consequences.

Having said this, it must be conceded that competitive sports are problematic at a time when it is necessary to change direction for the sake of future generations, other species and indeed all that makes it possible for diverse life on the planet to thrive. This is because the collectively shared mental template fuelling enthusiasm for sport is perfectly congruent with the mentality (or discourse, if you prefer) that makes the task of shifting gear so daunting. *Citius, altius, fortius* is exactly what the world needs less of. Now recall that the trees in the hills surrounding Oslo need to grow to 30 m in order to reproduce, although the energy needed to achieve that height is largely wasted. One might by rights ask why on earth those trees cannot just get together and decide that as from next year, no tree is allowed to reach more than 5 m. Those who violate the principle will be executed by chainsaw and exported to Trafalgar Square as Christmas trees.

The short response to this proposition is that as far as we know, trees are incapable of making this kind of collective decision; but we human beings definitely are. That is why this era is called the Anthropocene and not the Dendrocene. So: Why do we humans continue to grow to 30 m when we are perfectly aware that four or five would have been adequate?

Put differently, we humans have a choice, and here lie our privilege and our damnation, since self-consciousness and morality make us responsible for our actions. Perhaps, considering that treadmill competition will be with us forever, alternative niches for competing could be created or strengthened, which leave no ecological footprint and enable us to cool down, scale down and slow down. A shining example to be followed by others could be the annual Danish pipe-smoking competitions. The aim at these events is to keep two grammes of tobacco glowing for as long as possible. The slowest participant wins. A noble sport indeed.

But then again, it is also perfectly possible to settle for chess, which has become a surprisingly popular spectator sport in recent years. At least in Norway. There is reason to suspect that this has something to do with the fact that the Norwegian Magnus Carlsen does really well in international chess tournaments. Nationalism has its own rewards and its own treacherous treadmills. But that is another storey.

## Data Availability Statement

The original contributions presented in the study are included in the article/supplementary material, further inquiries can be directed to the corresponding author.

## Author Contributions

The author confirms being the sole contributor of this work and has approved it for publication.

## Conflict of Interest

The author declares that the research was conducted in the absence of any commercial or financial relationships that could be construed as a potential conflict of interest.
